# Supplementary material for: Resource availability enhances positive tree functional diversity effects on carbon and nitrogen accrual in natural forests
Source: Nat Commun. 2024 Oct 4;15:8615. doi: 10.1038/s41467-024-53004-y (PMC11452543; doi:10.1038/s41467-024-53004-y)
Supplement: Supplementary file 1 — Supplementary Information [file 41467_2024_53004_MOESM1_ESM.pdf]

Supplementary Information

Supplementary Table 1. Effects of tree functional diversity (FD_{is}) and functional identity (CWM), stand age (SA), decadal mean annual solar radiation (Solar), climate moisture index (CMI), decadal cumulative of N deposition (ND), organic horizon soil C/N (OCN), heatwave intensity (HI) and soil pH on C accumulation in tree biomass.

Fixed effect	Coefficient	*F* value	*P*	R^2	VIF
**Most parsimonious model for Solar, CMI and ND(1) (R^2 = 0.15)**					
Solar	0.64	22.45	**0.000003**	0.05	1.50
CMI	-0.01	0.81	0.370	< 0.01	2.39
SA	0.004	6.72	**0.010**	0.01	1.30
FD_{is}	0.46	7.42	**0.007**	0.02	1.42
CWM_{PC1}	0.26	9.91	**0.002**	0.02	1.51
CWM_{PC2}	-0.04	0.03	0.858	< 0.01	1.47
FD_{is} × Solar	0.55	7.54	**0.006**	0.02	1.21
CWM_{PC2} × Solar	0.24	4.30	**0.039**	0.01	1.80
CWM_{PC2} × CMI	-0.01	6.93	**0.009**	0.02	1.94
**Most parsimonious model for OCN(2) (R^2 = 0.17)**					
OCN	-0.66	14.91	**0.0001**	0.05	1.55
SA	0.45	12.32	**0.0005**	0.04	1.12
FD_{is}	0.32	11.94	**0.0006**	0.04	1.28
CWM_{PC2}	0.04	0.21	0.673	< 0.01	1.08
FD_{is} × OCN	-0.47	8.30	**0.004**	0.03	1.32
CWM_{PC2} ×OCN	-0.29	5.52	**0.019**	0.02	1.06
**Most parsimonious model for HI(3) (R^2 = 0.16)**					
HI	-0.01	4.39	**0.037**	0.01	1.16
SA	0.002	3.25	0.072	0.01	1.07
FD_{is}	0.79	9.79	**0.002**	0.03	1.46
CWM_{PC1}	0.26	12.93	**0.0004**	0.04	1.48
FD_{is} × HI	0.02	2.87	0.091	0.01	1.45
CWM_{PC1} × HI	-0.01	5.87	**0.016**	0.02	1.45
**Most parsimonious model for soil pH(4) (R^2 = 0.11)**					
pH	-0.29	6.47	**0.012**	0.03	1.07
SA	0.005	6.06	**0.014**	0.02	1.06
FD_{is}	0.64	6.91	**0.009**	0.03	1.30
CWM_{PC1}	0.35	14.36	**0.0002**	0.06	1.33
FD_{is} × pH	-0.33	2.88	0.091	0.01	1.03

VIF: variance inflation factor; FD_{is}: functional diversity; CWM_{PC1} & CWM_{PC2}: community-weighted mean of trait values. Higher CWM_{PC1} values indicate traits associated with a high

acquisitive strategy, whereas lower values indicate a lower acquisitive strategy. Higher CWM_{PC2} values indicate traits associated with a lower maximum tree height (see Supplementary Fig. 7). The significance (*P*) is reported for each term tested, with *P* values calculated using a one-sided F test. The P values that are less than 0.05 are highlighted in bold.

(1) $\Delta C_{Tree} = \beta_0 + \beta_1 \cdot SA + \beta_2 \cdot Solar + \beta_3 \cdot CMI + \beta_4 \cdot FD_{is} + \beta_5 \cdot CWM_{PC1} + \beta_6 \cdot CWM_{PC2} + \beta_7 \cdot Solar \times FD_{is} + \beta_8 \cdot Solar \times CWM_{PC2} + \beta_9 \cdot CMI \times CWM_{PC2} + \varepsilon$

(2) $\Delta C_{Tree} = \beta_0 + \beta_1 \cdot SA + \beta_2 \cdot OCN + \beta_3 \cdot FD_{is} + \beta_4 \cdot CWM_{PC2} + \beta_5 \cdot OCN \times FD_{is} + \beta_6 \cdot OCN \times CWM_{PC2} + \varepsilon$

(3) $\Delta C_{Tree} = \beta_0 + \beta_1 \cdot SA + \beta_2 \cdot HI + \beta_3 \cdot FD_{is} + \beta_4 \cdot CWM_{PC1} + \beta_5 \cdot HI \times FD_{is} + \beta_6 \cdot HI \times CWM_{PC1} + \varepsilon$

(4) $\Delta C_{Tree} = \beta_0 + \beta_1 \cdot SA + \beta_2 \cdot pH + \beta_3 \cdot FD_{is} + \beta_4 \cdot CWM_{PC1} + \beta_5 \cdot pH \times FD_{is} + \varepsilon$

Supplementary Table 2. Effects of tree functional diversity (FD_{is}), functional identity (CWM), stand age (SA), decadal mean annual solar radiation (Solar), climate moisture index (CMI), decadal cumulative of N deposition (ND), organic horizon soil C/N (OCN) and heatwave intensity (HI) on C accumulation in the organic soil horizon.

Fixed effect	Coefficient	*F* value	*P*	R^2	VIF
**Most parsimonious model for Solar, CMI and ND[(1)] (R^2 = 0.11)**					
CMI	-0.003	2.97	0.086	< 0.01	1.79
ND	-0.13	4.00	**0.047**	0.01	1.34
FD_{is}	0.90	2.99	0.085	< 0.01	1.31
CWM_{PC1}	-0.26	0.001	0.973	< 0.01	1.95
CWM_{PC2}	0.18	2.85	0.092	< 0.01	1.39
$FD_{is} \times$ CMI	0.04	13.20	**0.0003**	0.03	1.18
$CWM_{PC1} \times$ CMI	-0.02	12.49	**0.0005**	0.03	1.41
$CWM_{PC1} \times$ ND	0.11	4.41	**0.036**	0.02	1.60
$CWM_{PC2} \times$ CMI	0.01	8.90	**0.003**	0.02	1.49
**Most parsimonious model for OCN[(2)] (R^2 = 0.10)**					
OCN	-0.02	0.46	0.498	< 0.01	2.67
FD_{is}	1.13	4.63	**0.032**	0.02	1.68
CWM_{PC1}	-0.30	3.11	0.079	0.01	4.99
CWM_{PC2}	0.02	0.45	0.503	< 0.01	1.39
$FD_{is} \times$ OCN	0.07	3.76	0.053	0.01	1.74
$CWM_{PC1} \times$ OCN	-0.05	8.14	**0.005**	0.02	3.82
$CWM_{PC2} \times$ OCN	0.05	15.76	**0.00009**	0.04	1.31
**Most parsimonious model for HI[(3)] (R^2 = 0.04)**					
HI	-0.005	0.33	0.564	< 0.01	1.27
FD_{is}	0.24	1.09	0.298	< 0.01	1.04
CWM_{PC2}	0.05	2.24	0.135	< 0.01	1.79
$FD_{is} \times$ HI	-0.04	8.52	**0.004**	0.02	1.04
$CWM_{PC2} \times$ HI	-0.02	3.37	0.067	0.01	1.50

VIF: variance inflation factor; FD_{is}: functional diversity; CWM_{PC1} & CWM_{PC2}: community-weighted mean of trait values. Higher CWM_{PC1} values indicate traits associated with a high acquisitive strategy, whereas lower values indicate a lower acquisitive strategy. Higher CWM_{PC2} values indicate traits associated with a lower maximum tree height (see Supplementary Fig. 7). The significance (*P*) is reported for each term tested, with *P* values calculated using a one-sided F test. The P values that are less than 0.05 are highlighted in bold.

[1)] $\Delta C_{Organic} = \beta_0 + \beta_1 \cdot SA + \beta_2 \cdot CMI + \beta_3 \cdot ND + \beta_4 \cdot FD_{is} + \beta_5 \cdot CWM_{PC1} + \beta_6 \cdot CWM_{PC2} + \beta_7 \cdot CMI \times FD_{is} + \beta_8 \cdot CMI \times CWM_{PC1} + \beta_9 \cdot CMI \times CWM_{PC2} + \beta_{10} \cdot ND \times CWM_{PC1} + \varepsilon$

[(2)] $\Delta C_{Organic} = \beta_0 + \beta_1 \cdot OCN + \beta_2 \cdot FD_{is} + \beta_3 \cdot CWM_{PC1} + \beta_4 \cdot CWM_{PC2} + \beta_5 \cdot OCN \times FD_{is} + \beta_6 \cdot OCN \times CWM_{PC1} + \beta_7 \cdot OCN \times CWM_{PC2} + \varepsilon$

[(3)] $\Delta C_{Organic} = \beta_0 + \beta_1 \cdot HI + \beta_2 \cdot FD_{is} + \beta_3 \cdot CWM_{PC2} + \beta_4 \cdot HI \times FD_{is} + \beta_5 \cdot HI \times CWM_{PC2} + \varepsilon$

Supplementary Table 3. Effects of tree functional diversity (FD_{is}), functional identity (CWM), stand age (SA), decadal mean annual solar radiation (Solar), climate moisture index (CMI), decadal cumulative of N deposition (ND), organic horizon soil C/N (OCN) and mineral horizon soil C/N (MCN) on C accumulation in the mineral soil horizon.

Fixed effect	Coefficient	*F* value	*P*	R^2	VIF
**Most parsimonious model for Solar, CMI and ND[(1)] (R^2 = 0.11)**					
Solar	0.22	2.56	0.111	0.01	1.36
ND	-0.16	3.83	0.051	0.01	1.67
FD_{is}	0.62	6.69	**0.010**	0.02	1.07
CWM_{PC2}	0.35	9.44	**0.002**	0.03	1.21
FD_{is} × ND	0.32	9.29	**0.003**	0.03	1.06
**Most parsimonious model with OCN[(2)] (R^2 = 0.10)**					
OCN	-0.0004	0.74	0.391	< 0.01	1.94
SA	0.003	2.28	0.133	< 0.01	1.22
FD_{is}	0.26	1.90	0.170	< 0.01	1.43
CWM_{PC1}	0.25	1.22	0.270	< 0.01	3.04
CWM_{PC2}	0.54	14.84	**0.0002**	0.04	1.42
FD_{is} × OCN	-0.07	4.02	**0.046**	0.01	1.50
CWM_{PC1} × OCN	0.04	9.41	**0.002**	0.03	2.74
**Most parsimonious model with MCN[(3)] (R^2 = 0.09)**					
MCN	-0.01	1.71	0.193	< 0.01	1.75
FD_{is}	0.66	6.64	**0.011**	0.02	1.29
CWM_{PC1}	-0.12	3.52	0.062	0.01	1.64
CWM_{PC2}	0.38	12.29	**0.0005**	0.04	1.07
FD_{is} × MCN	-0.03	2.80	0.096	0.01	1.18
CWM_{PC1} × MCN	0.01	2.47	0.117	0.01	1.82

VIF: variance inflation factor; FD_{is}: functional diversity; CWM_{PC1} & CWM_{PC2}: community-weighted mean of trait values. Higher CWM_{PC1} values indicate traits associated with a high acquisitive strategy, whereas lower values indicate a lower acquisitive strategy. Higher CWM_{PC2} values indicate traits associated with a lower maximum tree height (see Supplementary Fig. 7). The significance (*P*) is reported for each term tested, with *P* values calculated using a one-sided F test. The P values that are less than 0.05 are highlighted in bold.

(1) $\Delta C_{Mineral} = \beta_0 + \beta_1 \cdot Solar + \beta_2 \cdot ND + \beta_3 \cdot FD_{is} + \beta_4 \cdot CWM_{PC2} + \beta_5 \cdot ND \times FD_{is} + \varepsilon$

(2) $\Delta C_{Mineral} = \beta_0 + \beta_1 \cdot OCN + \beta_2 \cdot FD_{is} + \beta_3 \cdot CWM_{PC1} + \beta_4 \cdot CWM_{PC2} + \beta_5 \cdot OCN \times FD_{is} + \beta_6 \cdot OCN \times CWM_{PC1} + \varepsilon$

(3) $\Delta C_{Mineral} = \beta_0 + \beta_1 \cdot MCN + \beta_2 \cdot FD_{is} + \beta_3 \cdot CWM_{PC1} + \beta_4 \cdot CWM_{PC2} + \beta_5 \cdot MCN \times FD_{is} + \beta_6 \cdot MCN \times CWM_{PC1} + \varepsilon$

Supplementary Table 4. Effects of tree functional diversity (FD_{is}), functional identity (CWM), stand age (SA), decadal mean annual solar radiation (Solar), climate moisture index (CMI), decadal cumulative of N deposition (ND), and heatwave intensity (HI) on N accumulation in organic and mineral soil horizon.

Fixed effect	Coefficient	*F* value	*P*	R^2	VIF
**Most parsimonious model of ΔOrganic horizon N for Solar, CMI and ND(1) (R^2 = 0.05)**					
CMI	-0.0003	0.65	0.422	< 0.01	1.31
ND	-0.01	5.78	**0.017**	0.01	1.23
FD_{is}	0.02	0.99	0.321	< 0.01	1.31
CWM_{PC1}	-0.001	2.35	0.126	0.01	1.87
FD_{is} × CMI	0.001	2.34	0.127	< 0.01	1.17
CWM_{PC1} × CMI	-0.001	5.85	**0.016**	0.01	1.36
CWM_{PC1} × ND	0.005	5.21	**0.023**	0.01	1.42
**Most parsimonious model of ΔOrganic horizon N for HI(2) (R^2 = 0.02)**					
HI	-0.0001	0.02	0.881	< 0.01	1.03
FD_{is}	-0.001	0.16	0.693	< 0.01	1.25
CWM_{PC1}	0.01	2.23	0.136	< 0.01	1.29
FD_{is} × HI	-0.002	7.25	**0.007**	0.02	1.07
**Most parsimonious model of ΔMineral horizon N for Solar, CMI and ND(3) (R^2 = 0.19)**					
Solar	0.01	4.91	**0.028**	0.01	1.65
CMI	0.0003	1.00	0.319	< 0.01	1.27
ND	-0.01	13.85	**0.0002**	0.04	1.85
FD_{is}	0.05	11.66	**0.0008**	0.03	1.34
CWM_{PC1}	-0.01	1.13	0.288	< 0.01	1.51
CWM_{PC2}	0.02	10.17	**0.002**	0.03	1.31
FD_{is} × ND	0.02	10.28	**0.002**	0.03	1.40
FD_{is} × Solar	-0.02	3.21	0.075	0.01	1.58
CWM_{PC1} × CMI	0.0004	7.57	**0.006**	0.02	1.30
**Most parsimonious model of ΔMineral horizon N for HI(4) (R^2 = 0.12)**					
HI	-0.00004	0.23	0.632	< 0.01	1.32
FD_{is}	0.05	11.37	**0.0009**	0.04	1.38
CWM_{PC1}	-0.01	3.33	0.069	0.01	1.46
CWM_{PC2}	0.03	10.82	**0.001**	0.03	1.89
FD_{is} × HI	0.001	3.85	0.051	0.01	1.14
CWM_{PC2} × HI	0.001	9.81	**0.002**	0.03	1.76

VIF: variance inflation factor; FD_{is}: functional diversity; CWM_{PC1} & CWM_{PC2}: community-weighted mean of trait values. Higher CWM_{PC1} values indicate traits associated with a high acquisitive strategy, whereas lower values indicate a lower acquisitive strategy. Higher CWM_{PC2}

values indicate traits associated with a lower maximum tree height (see Supplementary Fig. 7). The significance (P) is reported for each term tested, with P values calculated using a one-sided F test. The P values that are less than 0.05 are highlighted in bold.

(1) $\Delta N_{organic} = \beta_0 + \beta_1 \cdot CMI + \beta_2 \cdot ND + \beta_3 \cdot FD_{is} + \beta_4 \cdot CWM_{PC1} + \beta_5 \cdot CMI \times FD_{is} + \beta_6 \cdot CMI \times CWM_{PC1} + \beta_7 \cdot ND \times CWM_{PC1} + \varepsilon$

(2) $\Delta N_{organic} = \beta_0 + \beta_1 \cdot HI + \beta_2 \cdot FD_{is} + \beta_3 \cdot CWM_{PC1} + \beta_4 \cdot HI \times FD_{is} + \varepsilon$

(3) $\Delta N_{mineral} = \beta_0 + \beta_1 \cdot Solar + \beta_2 \cdot CMI + \beta_3 \cdot ND + \beta_4 \cdot FD_{is} + \beta_5 \cdot CWM_{PC1} + \beta_6 \cdot CWM_{PC2} + \beta_7 \cdot ND \times FD_{is} + \beta_8 \cdot Solar \times FD_{is} + \beta_9 \cdot CMI \times CWM_{PC1} + \varepsilon$

(4) $\Delta N_{mineral} = \beta_0 + \beta_1 \cdot HI + \beta_2 \cdot FD_{is} + \beta_3 \cdot CWM_{PC1} + \beta_4 \cdot CWM_{PC2} + \beta_5 \cdot HI \times FD_{is} + \beta_5 \cdot HI \times CWM_{PC2} + \varepsilon$

Supplementary Table 5. Effects of tree species evenness (Evenness) and its interaction with decadal mean annual solar radiation (Solar), climate moisture index (CMI), mean annual heatwave intensity (HI), decadal cumulative N deposition (ND), and soil pH on C accumulation in tree biomass and both C and N accumulation in the soil by replacing tree species evenness with functional diversity in Supplementary Tables 1, 2, 3 and 4.

Fixed effect	C accumulation			N accumulation		
	Coefficient	*P*	R^2	Coefficient	*P*	R^2
Model for resource gradient						
**Tree**						
Solar	0.66	**1.0×10^{-6}**	0.05			
Evenness	0.83	0.114	< 0.01			
Evenness × Solar	1.28	**0.010**	0.01			
**Organic horizon**						
CMI	-0.01	0.064	0.01	-0.0004	0.351	< 0.01
Evenness	2.26	**0.037**	0.01	0.08	0.072	< 0.01
Evenness × CMI	0.09	**0.0004**	0.03	0.002	0.078	< 0.01
**Mineral horizon**						
ND	-0.12	0.127	0.01	-0.01	**0.0003**	0.04
Evenness	1.29	0.295	< 0.01	0.11	**0.012**	0.02
Evenness × ND	1.21	**0.0004**	0.05	0.07	**4.5×10^{-5}**	0.05
Model for heatwave intensity						
**Tree**						
HI	-0.012	**0.022**	0.01			
Evenness	0.87	0.112	< 0.01			
Evenness × HI	-0.003	0.899	< 0.01			
**Organic horizon**						
HI	-0.004	0.570	< 0.01	-0.0001	0.934	< 0.01
Evenness	1.28	0.146	< 0.01	0.05	0.152	< 0.01
Evenness × HI	-0.10	**0.009**	**0.02**	-0.005	**0.002**	0.03
**Mineral horizon**						
HI				-0.0002	0.417	< 0.01
Evenness	0.61	0.373	< 0.01	0.09	**0.013**	0.02
Evenness × HI				0.003	0.056	0.01
Model for soil pH						
**Tree**						
pH	-0.27	**0.012**	0.09			
Evenness	0.68	0.288	< 0.01			
Evenness × pH	-0.87	0.103	0.01			

Solar: the mean annual solar radiation; CMI: the mean annual climate moisture index; ND: the decadal cumulative nitrogen deposition; HI: mean annual heatwave intensity; FD_{is}: functional

diversity; CWM_{PC1} & CWM_{PC2}: community-weighted mean of trait values. Higher CWM_{PC1} values indicate traits associated with a high acquisitive strategy, whereas lower values indicate a lower acquisitive strategy. Higher CWM_{PC2} values indicate traits associated with a lower maximum tree height (see Supplementary Fig. 7). The significance (*P*) is reported for each term tested, with *P* values calculated using a one-sided F test. The P values that are less than 0.05 are highlighted in bold.

Supplementary Table 6. Effects of tree species richness (Richness) and its interaction with decadal mean annual solar radiation (Solar), climate moisture index (CMI), mean annual heatwave intensity (HI), decadal cumulative N deposition (ND), and soil pH on C accumulation in tree biomass and both C and N accumulation in the soil by replacing tree species richness with functional diversity in Supplementary Tables 1, 2, 3 and 4.

Fixed effect	C accumulation			N accumulation		
	Coefficien	*P*	R^2	Coefficient	*P*	R^2
Model for resource gradient						
**Tree**						
Solar	0.59	**5.1×10^{-6}**	0.05			
Richness	0.35	**1.7×10^{-6}**	0.05			
Richness × Solar	0.13	0.102	< 0.01			
**Organic horizon**						
CMI	-0.01	0.065	< 0.01	-0.0004	0.396	< 0.01
Richness	0.16	0.239	< 0.01	0.004	0.561	< 0.01
Richness × CMI	0.001	0.742	< 0.01	-0.0001	0.426	< 0.01
**Mineral horizon**						
ND	-0.15	0.052	0.01	-0.02	**0.0002**	0.05
Richness	0.16	0.075	0.01	0.01	0.068	0.01
Richness × ND	0.02	0.580	< 0.01	0.002	0.471	< 0.01
Model for heatwave intensity						
**Tree**						
HI	-0.01	**0.042**	0.01			
Richness	0.46	**8.9×10^{-8}**	0.08			
Richness × HI	0.01	0.105	< 0.01			
**Organic horizon**						
HI	-0.01	0.474	< 0.01	-0.0001	0.843	< 0.01
Richness	-0.02	0.931	< 0.01	-0.004	0.528	< 0.01
Richness × HI	-0.01	**0.050**	0.01	-0.0004	0.084	< 0.01
**Mineral horizon**						
HI				-0.0002	0.387	< 0.01
Richness	0.15	0.135	< 0.01	0.007	0.176	< 0.01
Richness × HI				0.0001	0.593	< 0.01
Model for soil pH						
**Tree**						
pH	-0.20	0.051	0.02			
Richness	0.41	**2.7×10^{-6}**	0.10			
Richness × pH	-0.02	0.833	< 0.01			

Solar: the mean annual solar radiation; CMI: the mean annual climate moisture index; ND: the decadal cumulative nitrogen deposition; HI: mean annual heatwave intensity; FD_{is}: functional diversity; CWM_{PC1} & CWM_{PC2}: community-weighted mean of trait values. Higher CWM_{PC1}

values indicate traits associated with a high acquisitive strategy, whereas lower values indicate a lower acquisitive strategy. Higher CWM_{PC2} values indicate traits associated with a lower maximum tree height (see Supplementary Fig. 7). The significance (*P*) is reported for each term tested, with *P* values calculated using a one-sided F test. The P values that are less than 0.05 are highlighted in bold.

Supplementary Table 7. Summary statistics (mean, SD and range) of the permanent sample plots across Canada (2002–2018).

Attribute	Unit	Mean	SD	Range
Stand age	years	105.1	76.7	6 – 881
Long-term average of mean annual climate moisture index (CMI)	cm	33.6	37.8	-32.6 – 247.5
Long-term average of mean annual temperature (MAT)	℃	2.0	2.9	-4.9 – 9.4
Long-term average of solar radiation	MJ m^{-2}	11.4	1.1	7.8 – 14.6
Species richness	unitless	3.0	1.5	1 – 9
Species evenness	unitless	0.3	0.2	0 – 0.9
Functional diversity (functional dispersion, FD_{is})	unitless	0.7	0.6	0.00 – 2.4
Community weighted mean of resource acquisition traits (CWM_{PC1})	unitless	0.0	1.5	-1.8 – 5.2
Community weighted mean of resource colonization traits (CWM_{PC2})	unitless	0.0	1.1	-4.4 – 3.3
Measurement interval	years	9.7	2.9	3.0 – 15.2
Decadal tree biomass C stock change	kg m^{-2} $decade^{-1}$	1.7	2.5	0 – 2.6
Decadal Soil C stock change in the organic horizon	kg m^{-2} $decade^{-1}$	0.56	3.43	-23.32 – 13.34
Decadal Soil C stock change in 0-15 cm mineral horizon	kg m^{-2} $decade^{-1}$	0.24	2.08	-8.02 – 7.97
Decadal Soil N stock change in the organic horizon	kg m^{-2} $decade^{-1}$	0.02	0.14	-1.62 – 0.60
Decadal Soil N stock change in 0-15 cm mineral horizon	kg m^{-2} $decade^{-1}$	0.02	0.11	-0.65 – 0.29

Supplementary Table 8. Effects of tree functional diversity (FD_{is}), functional identity (CWM), decadal mean annual temperature (MAT), solar radiation (Solar) and climate moisture index (CMI), decadal cumulative N deposition (ND), and stand age (SA) on C accumulation in tree biomass and both C and N accumulation in the soil.

Fixed effect	Coefficient	*F* value	*P*	R^2
**Decadal tree C accumulation (R^2 = 0.15)**				
MAT	0.08	2.23	0.136	< 0.01
Solar	0.53	10.84	**0.001**	0.03
CMI	-0.01	1.94	0.165	< 0.01
SA	0.004	8.02	**0.005**	0.02
FD_{is}	0.41	5.90	**0.015**	0.02
CWM_{PC1}	0.25	9.08	**0.003**	0.03
CWM_{PC2}	0.03	0.25	0.616	< 0.01
FD_{is} × MAT	-0.07	0.65	0.419	< 0.01
FD_{is} × Solar	0.64	6.79	**0.009**	0.02
CWM_{PC2} × Solar	0.20	2.94	0.089	0.01
CWM_{PC2} × CMI	-0.01	6.85	**0.009**	0.02
**Decadal soil C accumulation in the organic horizon (R^2 = 0.11)**				
MAT	-0.04	0.14	0.710	< 0.01
CMI	-0.002	0.81	0.368	< 0.01
ND	-0.10	1.72	0.191	< 0.01
FD_{is}	0.86	3.35	0.068	< 0.01
CWM_{PC1}	-0.23	0.01	0.934	< 0.01
CWM_{PC2}	0.14	2.62	0.107	< 0.01
FD_{is} × MAT	0.07	0.24	0.624	< 0.01
FD_{is} × CMI	0.04	9.72	**0.002**	0.02
CWM_{PC1} × CMI	-0.02	11.96	**0.0006**	0.03
CWM_{PC1} × ND	0.10	2.93	0.088	< 0.01
CWM_{PC2} × CMI	0.01	8.95	**0.003**	0.02
**Decadal soil C accumulation in the mineral horizon (R^2 = 0.11)**				
MAT	0.002	<0.0001	1.000	< 0.01
Solar	0.21	2.08	0.151	< 0.01
ND	-0.16	3.20	0.075	0.01
FD_{is}	0.62	6.54	**0.011**	0.03
CWM_{PC2}	0.34	7.43	**0.007**	0.03
FD_{is} × MAT	0.04	0.18	0.675	< 0.01
FD_{is} × ND	0.30	6.80	**0.010**	0.03
**Decadal soil N accumulation in the organic horizon (R^2 = 0.06)**				

MAT	-0.004	1.82	0.179	< 0.01
CMI	-0.0001	0.12	0.733	< 0.01
ND	-0.005	1.62	0.205	< 0.01
FD_{is}	0.02	1.50	0.222	< 0.01
CWM_{PC1}	0.003	3.26	0.072	0.01
FD_{is} × MAT	0.007	1.33	0.249	< 0.01
FD_{is} × CMI	0.0004	0.65	0.422	< 0.01
CWM_{PC1} × CMI	-0.001	5.97	**0.015**	0.02
CWM_{PC1} × ND	0.003	2.05	0.153	< 0.01
**Decadal soil N accumulation in the mineral horizon (R^2 = 0.19)**				
MAT	-0.005	2.13	0.146	< 0.01
Solar	0.02	6.38	**0.012**	0.02
CMI	0.0005	1.77	0.185	< 0.01
ND	-0.01	7.43	**0.007**	0.03
FD_{is}	0.05	13.59	**0.0003**	0.05
CWM_{PC1}	-0.01	1.06	0.305	< 0.01
CWM_{PC2}	0.01	4.28	**0.040**	0.01
FD_{is} × Solar	-0.02	1.70	0.194	< 0.01
FD_{is} × MAT	-0.002	0.17	0.684	< 0.01
FD_{is} × ND	0.02	9.60	**0.002**	0.03
CWM_{PC1} × CMI	0.0005	8.87	**0.003**	0.03

Solar: mean annual solar radiation; MAT: mean annual temperature; CMI: mean annual climate moisture index; ND: the decadal cumulative nitrogen deposition; FD_{is}: functional diversity; CWM_{PC1} & CWM_{PC2}: community-weighted mean of trait values. Higher CWM_{PC1} values indicate traits associated with a higher acquisitive strategy, whereas lower values indicate a lower acquisitive strategy. Higher CWM_{PC2} values indicate traits associated with a lower maximum tree height (see Supplementary Fig. 7). The significance (*P*) is reported for each term tested, with *P* values calculated using a one-sided F test. The P values that are less than 0.05 are highlighted in bold.

Supplementary Table 9. Interaction effects of tree functional diversity (FD_{is}) and biome type (temperate and boreal forests) on C accumulation in tree biomass and both C and N accumulation in the soil.

Fixed effect	*F* value	*P*	R^2
**Decadal tree C accumulation**			
Biome type	0.66	0.416	< 0.01
FD_{is}	6.28	**0.013**	0.02
FD_{is} × Biome type	0.07	0.798	< 0.01
**Decadal soil C accumulation in the organic horizon**			
Biome type	0.04	0.849	< 0.01
FD_{is}	3.56	0.060	< 0.01
FD_{is} × Biome type	0.01	0.919	< 0.01
**Decadal soil C accumulation in the mineral horizon**			
Biome type	0.57	0.450	< 0.01
FD_{is}	7.08	**0.008**	0.03
FD_{is} × Biome type	0.19	0.662	< 0.01
**Decadal soil N accumulation in the organic horizon**			
Biome type	0.21	0.650	< 0.01
FD_{is}	1.20	0.275	< 0.01
FD_{is} × Biome type	0.01	0.919	< 0.01
**Decadal soil N accumulation in the mineral horizon**			
Biome type	2.89	0.091	0.01
FD_{is}	13.46	**0.0003**	0.04
FD_{is} × Biome type	0.24	0.626	< 0.01

The significance (*P*) is reported for each term tested, with *P* values calculated using a one-sided F test. The P values that are less than 0.05 are highlighted in bold.

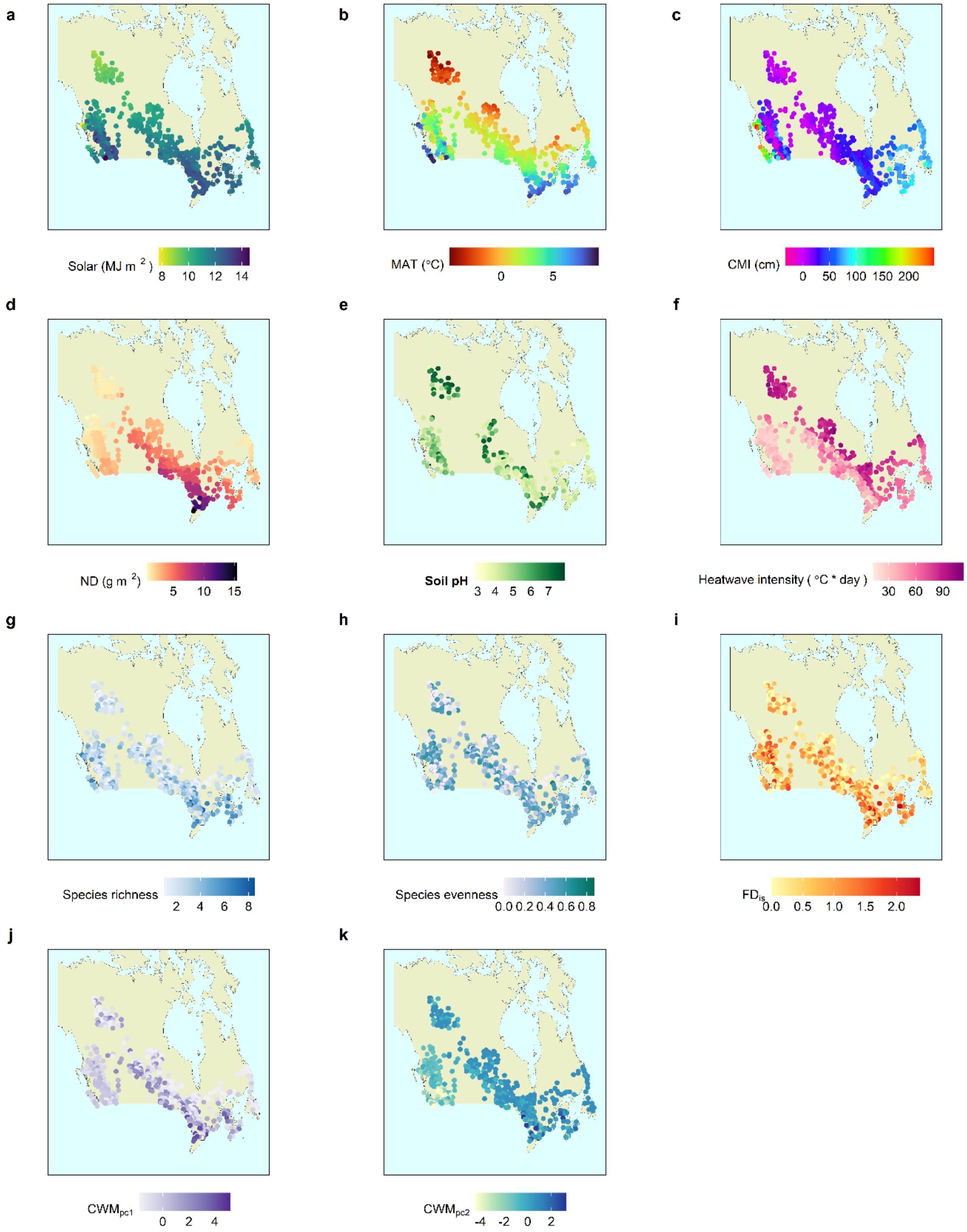
a
Solar (MJ m 2)
8 10 12 14
b
MAT (°C)
0 5
c
CMI (cm)
0 50 100 150 200
d
ND (g m 2)
5 10 15
e
Soil pH
3 4 5 6 7
f
Heatwave intensity (°C * day)
30 60 90
g
Species richness
2 4 6 8
h
Species evenness
0.0 0.2 0.4 0.6 0.8
i
FDis
0.0 0.5 1.0 1.5 2.0
j
CWMpc1
0 2 4
k
CWMpc2
-4 -2 0 2

Supplementary Fig. 1 The distribution of 513 ground plots from the Canadian National Forest Inventory (NFI) with tree carbon accumulation measurements. Solar: mean annual solar radiation; MAT: mean annual temperature; CMI: mean annual climate moisture index; ND: the decadal cumulative nitrogen deposition; FD_{is}: functional diversity; CWM_{PC1} & CWM_{PC2}: community-weighted mean of trait values. Higher CWM_{PC1} values indicate traits associated with a high acquisitive strategy, whereas lower values indicate a low acquisitive strategy. Higher CWM_{PC2} values indicate traits associated with a lower maximum tree height.

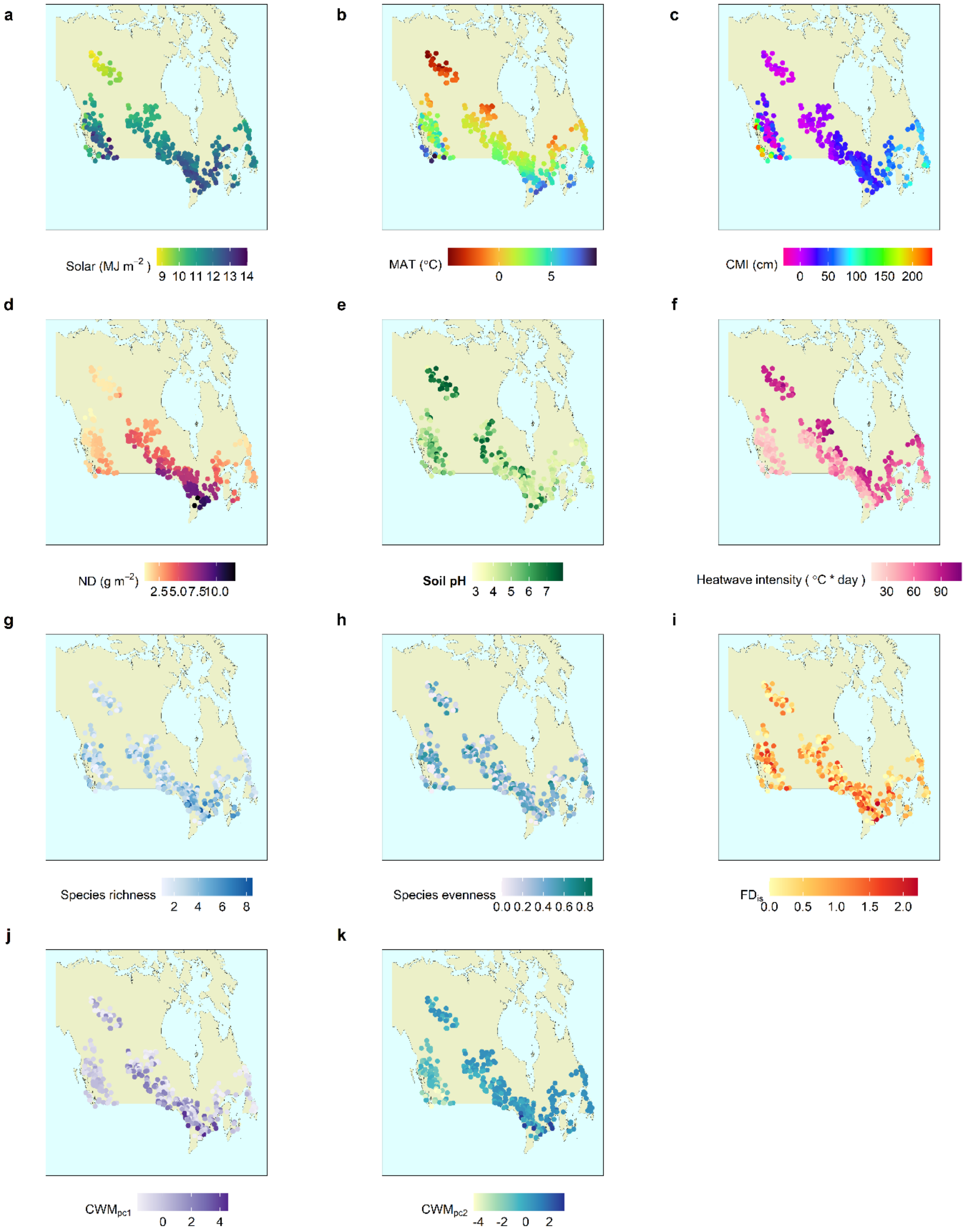
a
Solar (MJ m^{-2})
9 10 11 12 13 14
b
MAT (°C)
0 5
c
CMI (cm)
0 50 100 150 200
d
ND (g m^{-2})
2.55.07.510.0
e
Soil pH
3 4 5 6 7
f
Heatwave intensity (°C * day)
30 60 90
g
Species richness
2 4 6 8
h
Species evenness
0.0 0.2 0.4 0.6 0.8
i
FD_{is}
0.0 0.5 1.0 1.5 2.0
j
CWM_{pc1}
0 2 4
k
CWM_{pc2}
-4 -2 0 2

Supplementary Fig. 2 The distribution of 360 ground plots from the Canadian National Forest Inventory (NFI) with organic soil horizon carbon and nitrogen accumulation measurements. Solar: mean annual solar radiation; MAT: mean annual temperature; CMI: mean annual climate moisture index; ND: the decadal cumulative nitrogen deposition; FD_{is}: functional diversity; CWM_{PC1} & CWM_{PC2}: community-weighted mean of trait values. Higher CWM_{PC1} values indicate traits associated with a high acquisitive strategy, whereas lower values indicate a low acquisitive strategy. Higher CWM_{PC2} values indicate traits associated with a lower maximum tree height.

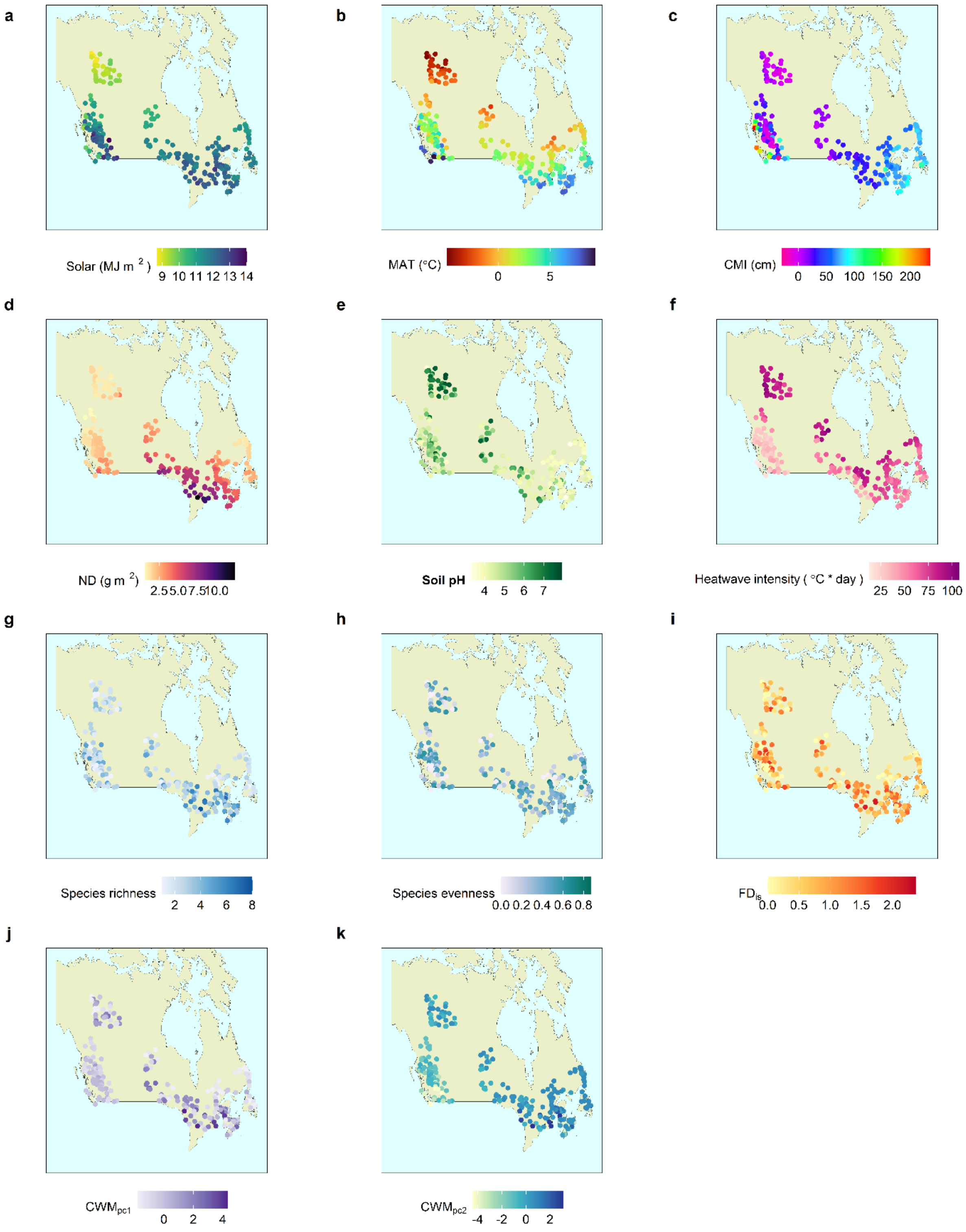

Supplementary Fig. 3 The distribution of 244 ground plots from the Canadian National Forest Inventory (NFI) with mineral soil horizon carbon and nitrogen accumulation measurements. Solar: mean annual solar radiation; MAT: mean annual temperature; CMI: mean annual climate moisture index; ND: the decadal cumulative nitrogen deposition; FD_{is}: functional diversity;

CWM_{PC1} & CWM_{PC2}: community-weighted mean of trait values. Higher CWM_{PC1} values indicate traits associated with a high acquisitive strategy, whereas lower values indicate a low acquisitive strategy. Higher CWM_{PC2} values indicate traits associated with a lower maximum tree height.

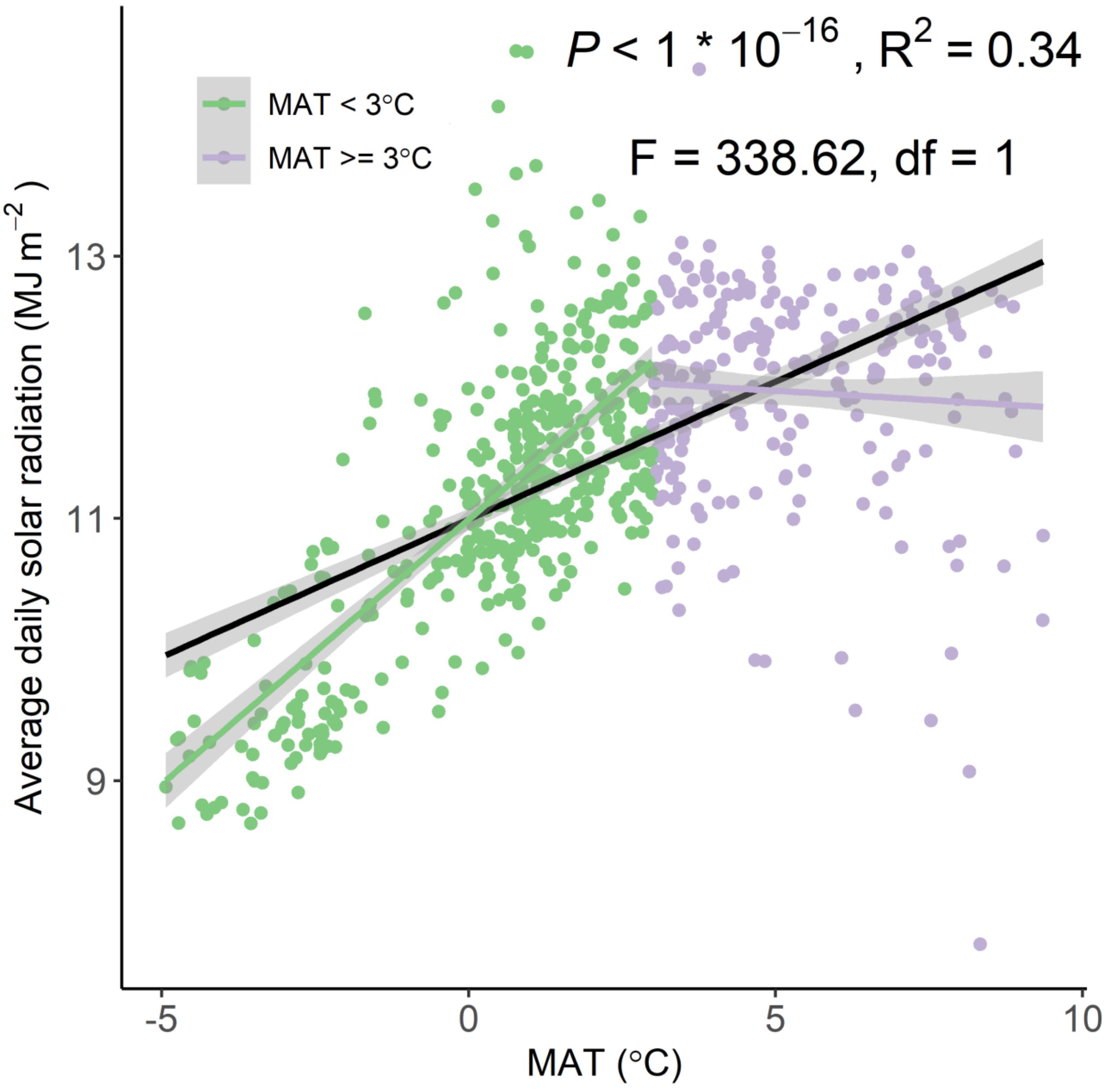

Supplementary Fig. 4 The MAT in relation to solar radiation. The black and coloured lines and grey shaded areas represent the fitted regression and its bootstrapped 95% confidence intervals. MAT: mean annual temperature. The *P* values calculated using a one-sided F test. df: degrees of freedom.

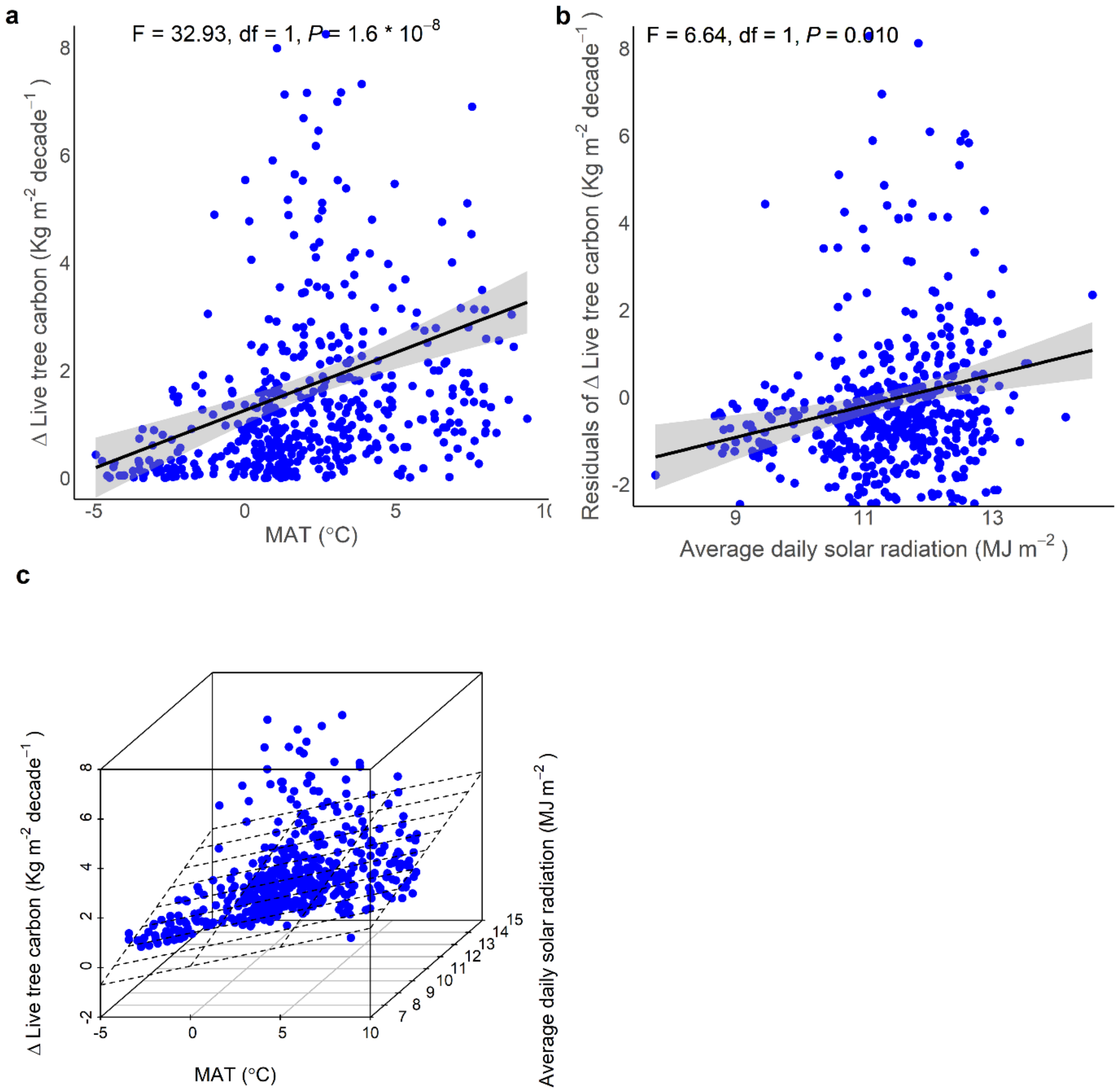

Supplementary Fig. 5 Relationships between tree C accumulation and mean annual temperature (MAT) and solar radiation. **a,** tree C accumulation in relation to MAT; **b,** residual tree C accumulation after removing MAT effect in relation to solar radiation; **c,** linear three-dimensional regression figure of tree C accumulation in relation to MAT and solar radiation. The black line and grey shaded areas represent the fitted regression and its bootstrapped 95% confidence intervals. The *P* values calculated using a one-sided F test. df: degrees of freedom.

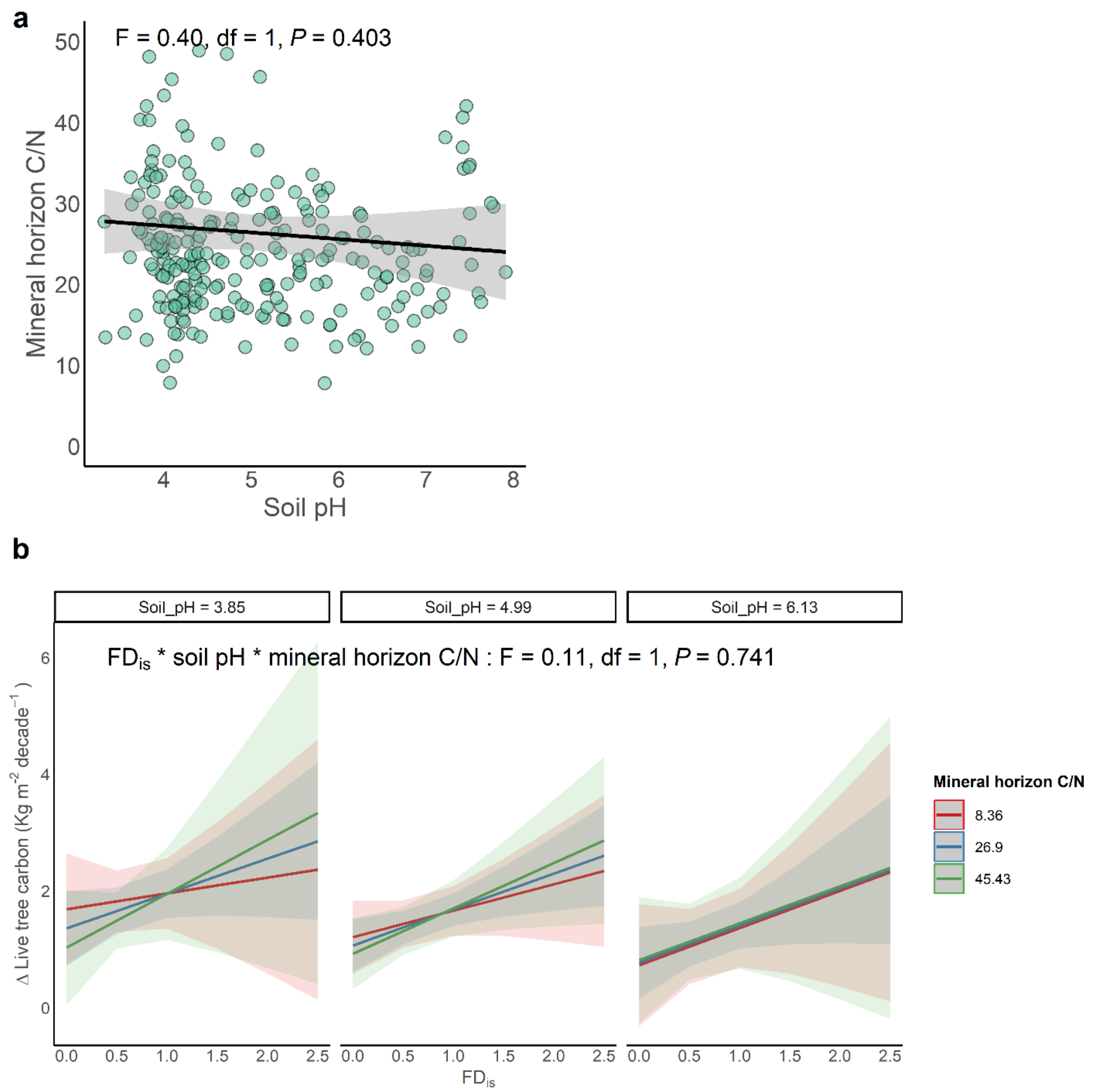

Supplementary Fig. 6 Relationships between soil pH and soil C/N ratios in the mineral horizon and soil pH-dependent response of tree C accumulation to functional diversity (FD_{is}) across different soil C/N ratio levels. The coloured line and grey shaded areas represent the fitted regression and its bootstrapped 95% confidence intervals. The *P* values calculated using a one-sided F test. df: degrees of freedom.

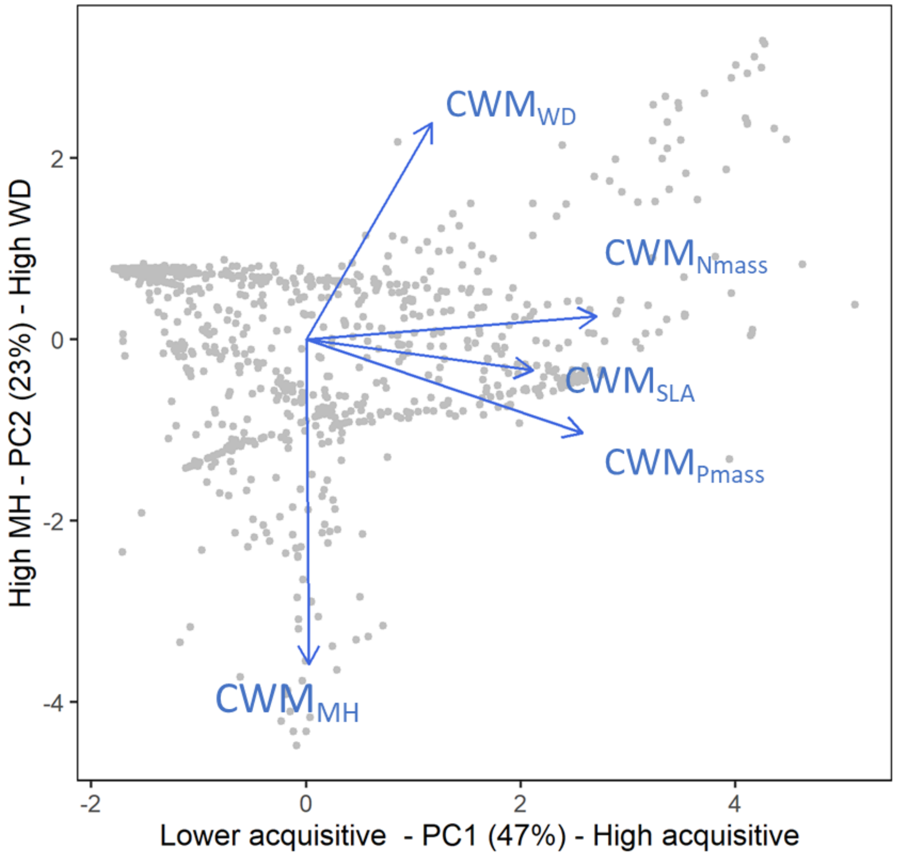

Supplementary Fig. 7 The result of principal component analysis (PCA) showing permanent sampling plots and each functional identity (community-weighted mean of trait values, CWM). CWM_{Nmass} = CWM of nitrogen content per leaf mass, CWM_{Pmass} = CWM of phosphorus content per leaf mass, CWM_{SLA} = CWM of specific leaf area, CWM_{WD} = CWM of wood density, CWM_{MH} = CWM of maximum height. The first axis (PC1) represents traits associated with acquisitive strategies, while the second axis (PC2) refers to traits associated with wood density (WD) versus the maximum height (MH) of trees. The graph was also used in Chen et al. Tree diversity increases decadal forest soil carbon and nitrogen accrual. Nature 618 (2023).

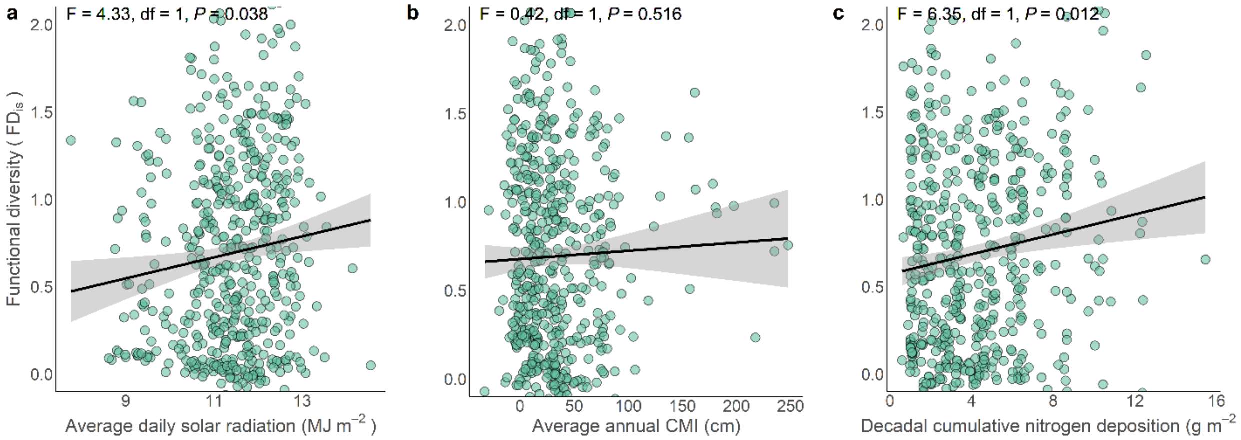

Supplementary Fig. 8 Relationships between tree functional diversity and decadal mean annual solar radiation, climate moisture index (CMI), decadal cumulative of nitrogen deposition. The black line and grey shaded areas represent the fitted regression and its bootstrapped 95% confidence intervals. The *P* values calculated using a one-sided F test. df: degrees of freedom.
