# Supplementary material for: Resource availability enhances positive tree functional diversity effects on carbon and nitrogen accrual in natural forests
Source: Nat Commun. 2024 Oct 4;15:8615. doi: 10.1038/s41467-024-53004-y (PMC11452543; doi:10.1038/s41467-024-53004-y)
Supplement: Supplementary file 3 — Reporting Summary [file 41467_2024_53004_MOESM3_ESM.pdf]

nature portfolio

Corresponding author(s): Scott Chang

Last updated by author(s): Sep 20, 2024

Reporting Summary

Nature Portfolio wishes to improve the reproducibility of the work that we publish. This form provides structure for consistency and transparency in reporting. For further information on Nature Portfolio policies, see our Editorial Policies and the Editorial Policy Checklist.

Statistics

For all statistical analyses, confirm that the following items are present in the figure legend, table legend, main text, or Methods section.

n/a	Confirmed	
☐	☒	The exact sample size (*n*) for each experimental group/condition, given as a discrete number and unit of measurement
☐	☒	A statement on whether measurements were taken from distinct samples or whether the same sample was measured repeatedly
☐	☒	The statistical test(s) used AND whether they are one- or two-sided<br>*Only common tests should be described solely by name; describe more complex techniques in the Methods section.*
☐	☒	A description of all covariates tested
☐	☒	A description of any assumptions or corrections, such as tests of normality and adjustment for multiple comparisons
☐	☒	A full description of the statistical parameters including central tendency (e.g. means) or other basic estimates (e.g. regression coefficient) AND variation (e.g. standard deviation) or associated estimates of uncertainty (e.g. confidence intervals)
☐	☒	For null hypothesis testing, the test statistic (e.g. *F*, *t*, *r*) with confidence intervals, effect sizes, degrees of freedom and *P* value noted<br>*Give P values as exact values whenever suitable.*
☒	☐	For Bayesian analysis, information on the choice of priors and Markov chain Monte Carlo settings
☒	☐	For hierarchical and complex designs, identification of the appropriate level for tests and full reporting of outcomes
☒	☐	Estimates of effect sizes (e.g. Cohen's *d*, Pearson's *r*), indicating how they were calculated

Our web collection on statistics for biologists contains articles on many of the points above.

Software and code

Policy information about availability of computer code

Data collection	We used plot-level data from the Canadian National Forest Inventory (NFI) database. The data was acquired through a contract between Antony Taylor and the Canadian Forest Service
Data analysis	The data analysis was conducted in R 4.3.1 (R Core Team, 2024), using packages including 'data.table (version 1.13.6)', 'MuMIn' (version 1.47.5), 'lme4 (version 1.1-26)', 'lmerTest (version 3.1-3)', 'ggplot2 (version 3.3.3)', 'maps (version 3.3.0)', "interactions" (version 1.1.5) and 'cowplot (version 1.1.1)'. Details were reported in statistical analysis section of the Methods. The code used in this study is available at Figshare (https://doi.org/10.6084/m9.figshare.25037213).

For manuscripts utilizing custom algorithms or software that are central to the research but not yet described in published literature, software must be made available to editors and reviewers. We strongly encourage code deposition in a community repository (e.g. GitHub). See the Nature Portfolio guidelines for submitting code & software for further information.

Data

Policy information about availability of data

All manuscripts must include a data availability statement. This statement should provide the following information, where applicable:

- Accession codes, unique identifiers, or web links for publicly available datasets
- A description of any restrictions on data availability
- For clinical datasets or third party data, please ensure that the statement adheres to our policy

The data on tree biomass carbon accumulation, soil carbon and nitrogen accumulation, and local environmental condition generated in this study have been deposited in the Figshare database under (https://doi.org/10.6084/m9.figshare.25037213). The raw tree and soil data are protected and are not available due to data privacy laws.

Research involving human participants, their data, or biological material

Policy information about studies with human participants or human data. See also policy information about sex, gender (identity/presentation), and sexual orientation and race, ethnicity and racism.

Reporting on sex and gender	We do not have any data involving human participants, their data, or biological material
Reporting on race, ethnicity, or other socially relevant groupings	We do not have any data involving human participants, their data, or biological material
Population characteristics	We do not have any data involving human participants, their data, or biological material
Recruitment	We do not have any data involving human participants, their data, or biological material
Ethics oversight	We do not have any data involving human participants, their data, or biological material

Note that full information on the approval of the study protocol must also be provided in the manuscript.

Field-specific reporting

Please select the one below that is the best fit for your research. If you are not sure, read the appropriate sections before making your selection.

☐ Life sciences ☐ Behavioural & social sciences ☒ Ecological, evolutionary & environmental sciences

For a reference copy of the document with all sections, see nature.com/documents/nr-reporting-summary-flat.pdf

Ecological, evolutionary & environmental sciences study design

All studies must disclose on these points even when the disclosure is negative.

Study description	In this study, we investigated the effects of resource availability and non-resource stress on the relationship between tree diversity and C and N accumulation in trees and forest soils by analyzing Canada's broadly distributed National Forest Inventory (NFI) plot network.
Research sample	We used plot-level data from the Canadian National Forest Inventory (NFI) database. Within each plot, tree species, DBH were recorded and soils from organic and mineral horizon were sampled.
Sampling strategy	No sample-size calculation were used. The plots were selected at random.
Data collection	In each plot, which comprises several sub-plots, a 'Large Tree Plot' was established with a radius of 11.28 m and an area of 400 m2 (0.04 ha). Within this Large Tree Plot, all canopy trees (tree stems ≥ 9.0 cm in diameter at breast height) were systematically numbered, tagged, identified for species, and measured for both height and DBH. In addition to the 'Large Tree Plot', four 1 m2 "Microplots" were established outside of the large tree plot (but within a 15 m radius of the Large Tree Plot centre). From each of these microplots, an organic horizon soil sample was collected that comprises the litter, fibric, and humus layers (over 17% organic C by mass) using 20 × 20 cm (inside dimensions) aluminum sampling frames. Furthermore, seven mineral soil horizon samples (less than 17% organic C) were collected from each NFI microplot at fixed depths (0–15 cm) using a 10-cm diameter auger. The collected organic and mineral soil samples were dried at 70 °C in an oven and were subsequently sieved using 8 mm and 2 mm screens to eliminate gravel and roots, respectively.
Timing and spatial scale	The NFI plots were established and monitored by Canadian provincial authorities between 2000 and 2006 (first measurement) and subsequently re-measured between 2008 and 2017 (second measurement) following the same standard ground sampling guidelines established by the Canadian Forest Inventory Committee. After excluding missing values for each horizon, 513 plots for canopy trees , 360 plots for organic soil horizon samples and 244 plots for 0-15 cm mineral soil horizon samples that span from 44°00′–64° 24′ N to 53°24′–128°36′ W were included in the statistical analyses

Data exclusions	For inclusion in our analysis, we selected only those plots situated in unmanaged forest stands at the time of sampling, with two measurements conducted and complete data coverage for forest canopy composition, stand age, tree C accumulation, soil C and N stocks.
Reproducibility	All attempts to repeat the analysis and results were successful.
Randomization	The data were analyzed with a random effect model. Bootstrapping sampling was used.
Blinding	Complete blinding in bootstrapping process.

Did the study involve field work? ☒ Yes ☐ No

Field work, collection and transport

Field conditions	After excluding missing values for each horizon, 513 plots for canopy trees , 360 plots for organic soil horizon samples and 244 plots for 0-15 cm mineral soil horizon samples that span from 44°00′–64°24′ N to 53°24′–128°36′ W were included in the statistical analyses . Weather conditions were shown in Supplementary Fig. 1, 2, 3
Location	The NFI database encompasses a network of permanent ground plots covering much of Canada's forests across boreal and temperate biomes, spanning from 44°00′–64°24′ N to 53°24′–128°36′ W
Access & import/export	We just used the data. This work was made possible only by long-term data-collection efforts of the Canadian National Forest Inventory team.
Disturbance	We just used the data. This work was made possible only by long-term data-collection efforts of the Canadian National Forest Inventory team.

Reporting for specific materials, systems and methods

We require information from authors about some types of materials, experimental systems and methods used in many studies. Here, indicate whether each material, system or method listed is relevant to your study. If you are not sure if a list item applies to your research, read the appropriate section before selecting a response.

Materials & experimental systems

n/a	Involved in the study	
☒	☐	Antibodies
☒	☐	Eukaryotic cell lines
☒	☐	Palaeontology and archaeology
☒	☐	Animals and other organisms
☒	☐	Clinical data
☒	☐	Dual use research of concern
☒	☐	Plants

Methods

n/a	Involved in the study	
☒	☐	ChIP-seq
☒	☐	Flow cytometry
☒	☐	MRI-based neuroimaging

Plants

Seed stocks	We do not have plant material
Novel plant genotypes	We do not have plant material
Authentication	We do not have plant material
